# A multi-subunit *Chlamydia* vaccine inducing neutralizing antibodies and strong IFN-γ^+^ CMI responses protects against a genital infection in minipigs

**DOI:** 10.1038/icb.2015.79

**Published:** 2015-09-22

**Authors:** Sarah Bøje, Anja Weinreich Olsen, Karin Erneholm, Jørgen Steen Agerholm, Gregers Jungersen, Peter Andersen, Frank Follmann

**Affiliations:** 1 Section of Veterinary Reproduction and Obstetrics, Department of Large Animal Sciences, Faculty of Health and Medical Sciences, University of Copenhagen, Frederiksberg C, Denmark; 2 Chlamydia Vaccine Research, Department of Infectious Disease Immunology, Statens Serum Institut, Copenhagen S, Denmark; 3 Immunology and Vaccinology, National Veterinary Institute, Technical University of Denmark, Frederiksberg C, Denmark

## Abstract

Chlamydia is the most widespread sexually transmitted bacterial disease and a prophylactic vaccine is highly needed. Ideally, this vaccine is required to induce a combined response of Th1 cell-mediated immune (CMI) response in concert with neutralizing antibodies. Using a novel Göttingen minipig animal model, we evaluated the immunogenicity and efficacy of a multi-subunit vaccine formulated in the strong Th1-inducing adjuvant CAF01. We evaluated a mixture of two fusion proteins (Hirep1 and CTH93) designed to promote either neutralizing antibodies or cell-mediated immunity, respectively. Hirep1 is a novel immunogen based on the variant domain (VD) 4 region from major outer membrane protein (MOMP) serovar (Sv) D, SvE and SvF, and CTH93 is a fusion molecule of three antigens (CT043, CT414 and MOMP). Pigs were immunized twice intramuscularly with either Hirep1+CTH93/CAF01, UV-inactivated *Chlamydia trachomatis* SvD bacteria (UV-SvD/CAF01) or CAF01. The Hirep1+CTH93/CAF01 vaccine induced a strong CMI response against the vaccine antigens and high titers of antibodies, particularly against the VD4 region of MOMP. Sera from Hirep1+CTH93/CAF01 immunized pigs neutralized *C. trachomatis* SvD and SvF infectivity *in vitro*. Both Hirep1+CTH93/CAF01 and UV-SvD/CAF01 vaccination protected pigs against a vaginal *C. trachomatis* SvD infection. In conclusion, the Hirep1+CTH93/CAF01 vaccine proved highly immunogenic and equally protective as UV-SvD/CAF01 showing promise for the development of a subunit vaccine against *Chlamydia*.

Chlamydia is the most common bacterial sexually transmitted disease, and worldwide, more than 100 million people are infected each year with the urogenital serovars (Svs) of *Chlamydia trachomatis*.^1^ The infection is often without symptoms and therefore left undiagnosed and untreated. This increases the risk of developing severe complications, such as pelvic inflammatory disease and infertility.^2^ At present, the main public intervention to reduce the incidence of genital chlamydia is routine screening programs, which until now has been largely unsuccessful,^1^ and development of a vaccine against chlamydia is therefore an international priority.^3^

In humans, repeated chlamydial infections are common, although natural immunity can develop.^4^ The exact immune correlates of protection are unknown, however, it is generally accepted that both humoral and cell-mediated immune (CMI) responses are important. The presence of antibodies in cervical secretions of women correlates with lower bacterial shedding,^5^ and interferon (IFN)-γ-positive responses in peripheral blood mononuclear cells (PBMCs) stimulated with cHsp60 correlates with a decreased risk of acquiring a subsequent chlamydial infection.^6^ Studies in mice consistently identify a key protective role for CD4^+^ Th1 cells and the cytokine IFN-γ.^7, 8, 9^ The protective role of antibodies is more multifaceted and ranges from direct bacterial neutralization^10^ to accelerating CMI through Fc-mediated uptake in antigen-presenting cells.^11^

Vaccines based on intact *Chlamydia* bacteria demonstrate that protection can be achieved both in non-human primates and in clinical trials.^12, 13^ However, protection seems to be Sv-specific^13^ and a whole-cell vaccine may potentially generate unwanted responses and lead to pathology,^14^ emphasizing the need for a broadly protective subunit vaccine. Vaccine research efforts have led to the identification of a large number of antigens with significant protective efficacy.^15, 16, 17, 18^ Efforts to identify a target for neutralizing antibodies have mainly focused on major outer membrane protein (MOMP), but the complex structure of the protein has complicated its use as a vaccine antigen.^19, 20^

In addition to identifying the right antigens, vaccine-induced immunity to *Chlamydia* highlights the challenge of identifying a clinically relevant delivery system that would induce a strong Th1 response, antibodies and long lived memory.^21^ The CAF01 adjuvant has demonstrated potent induction of CMI and humoral immunity with several chlamydial antigens in mouse models.^17, 22^ Importantly, the adjuvant has also been tested in phase I trials^23, 24^ with an excellent safety profile and is a promising adjuvant for a recombinant vaccine against *Chlamydia*.

So far, most *Chlamydia* vaccine research has been carried out in mice.^15, 16, 17^ To move promising vaccine candidates closer to a clinical trial, there is a need for testing in animal models with closer resemblance to humans than rodents. Pigs are immunologically and physiologically in many ways comparable with humans,^25, 26, 27^ facilitating the evaluation of vaccine immunogenicity in this animal species. Importantly, a previous study has demonstrated that pigs are susceptible to genital *C. trachomatis* infection.^28^

We recently constructed a novel recombinant version of MOMP, based on the variant domain (VD)4 regions of SvD, SvE and SvF (Hirep1) that induces high titered neutralizing antibodies, covering Svs causing up to 90% of all infections and showing for the first time that vaccine-induced antibodies can control *Chlamydia* infections.^29^ In the present study, we combine Hirep1 with a recombinant fusion molecule CTH93, consisting of previously identified antigens; CT043,^17, 30, 31^ MOMP (CT681)_amino acid (aa)34-371_^15, 16, 17, 31^ and CT414_aa605-840_. CTH93 represents a broad epitope repertoire covering both T- and B-cell epitopes, but lacks the ability to induce neutralizing antibodies. In the current study, we evaluate the immunogenicity and efficacy of Hirep1+CTH93 formulated in CAF01 in Göttingen minipigs.

## Results

### The Hirep1+CTH93/CAF01 vaccine is highly immunogenic

Göttingen minipigs were immunized with a mixture of Hirep1 (comprising the VD4 domain of MOMP SvD, SvE and SvF) and CTH93 (consisting of MOMP SvD_aa34-371_, CT043 and CT414_aa605-840_) (Supplementary Figure 1) formulated in CAF01, with the purpose of inducing neutralizing antibodies and strong T-cell responses. Throughout the study, the Hirep1+CTH93/CAF01-induced responses were compared with immune responses in UV-SvD/CAF01-immunized pigs. Nineteen sexually mature minipigs were randomly allocated into four immunization groups receiving 10 μg Hirep1+CTH93/CAF01, 100 μg Hirep1+CTH93/CAF01, 125 μg UV-SvD/CAF01 or CAF01 alone (Table 1). The pigs were immunized twice in the neck muscles spaced with an interval of 3 weeks.

**Figure 1:**
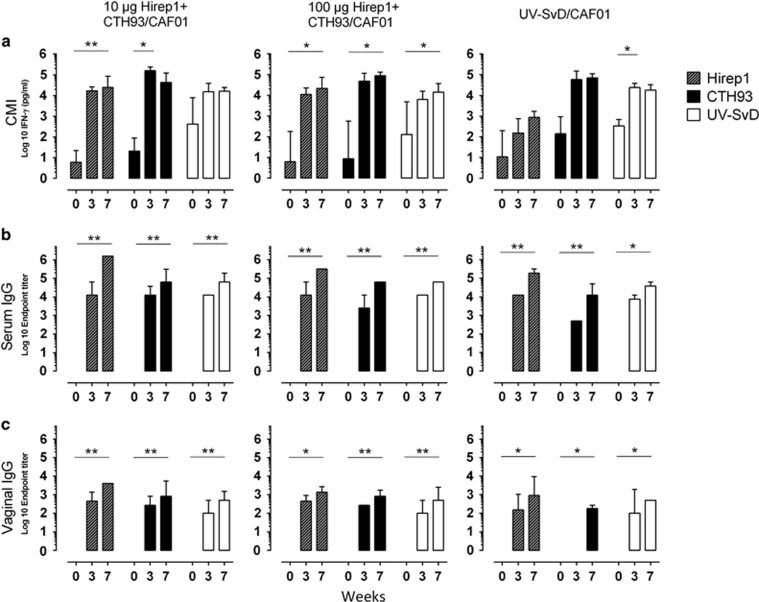
Characterization of vaccine-induced immune responses. Pigs were intramuscularly immunized at week 0 and 3 with 10 μg (*n*=5) or 100 μg (*n*=5) Hirep1+CTH93/CAF01 or UV-SvD/CAF01 (*n*=4). Blood samples and vaginal swabs were taken at weeks 0, 3 and 7 to evaluate CMI and humoral responses. (**a**) PBMCs were restimulated *in vitro* with Hirep1, CTH93 and UV-SvD. For each pig, the mean IFN-γ of triplicate culture wells was calculated and the bars represent the median±interquartile range within a group. (**b**) Serum IgG and (**c**) vaginal IgG titers specific for Hirep1, CTH93 and UV-SvD were analyzed in duplicates and the mean were calculated. Bars represent medians±interquartile range within a group. Statistical difference was calculated using the Friedman test followed by Dunn's multiple comparison test, *P<0.05, **P<0.01.

At week 0, 3 and 7, PBMCs were isolated from blood samples and restimulated with the recombinant, vaccine antigens (Hirep1 and CTH93) and with UV-SvD. After immunizations, Hirep1+CTH93/CAF01-immunized groups showed significant higher levels of IFN-γ against Hirep1 and CTH93 compared with week 0 (Figure 1a). Furthermore, the Hirep1+CTH93/CAF01 vaccine-induced CMI responses were able to recognize UV-SvD, comparable with the levels in UV-SvD/CAF01-immunized pigs.

To examine humoral immune responses, we collected sera and vaginal swabs at week 0, 3 and 7. Both groups of pigs immunized with Hirep1+CTH93/CAF01 had significant levels of specific IgG in serum (Figure 1b). The Hirep1+CTH93/CAF01-generated antibodies were able to recognize the surface of UV-SvD, comparable with the levels induced in UV-SvD/CAF01-immunized pigs. Importantly, high titers of vaccine-specific antibodies were also detected on the vaginal mucosa (Figure 1c), reflecting transudation of serum into the genital tract in the vaccinated pigs.

### Hirep1+CTH93/CAF01 vaccination primarily induces T-cell epitopes in the VD4 regions of MOMP from SvD, SvE and SvF

As there were no significant differences between immune responses in the groups receiving 10 and 100 μg Hirep1+CTH93/CAF01 (shown in Figure 1), we decided to pool results from the two hybrid vaccine groups for further analysis.

**Figure 2:**
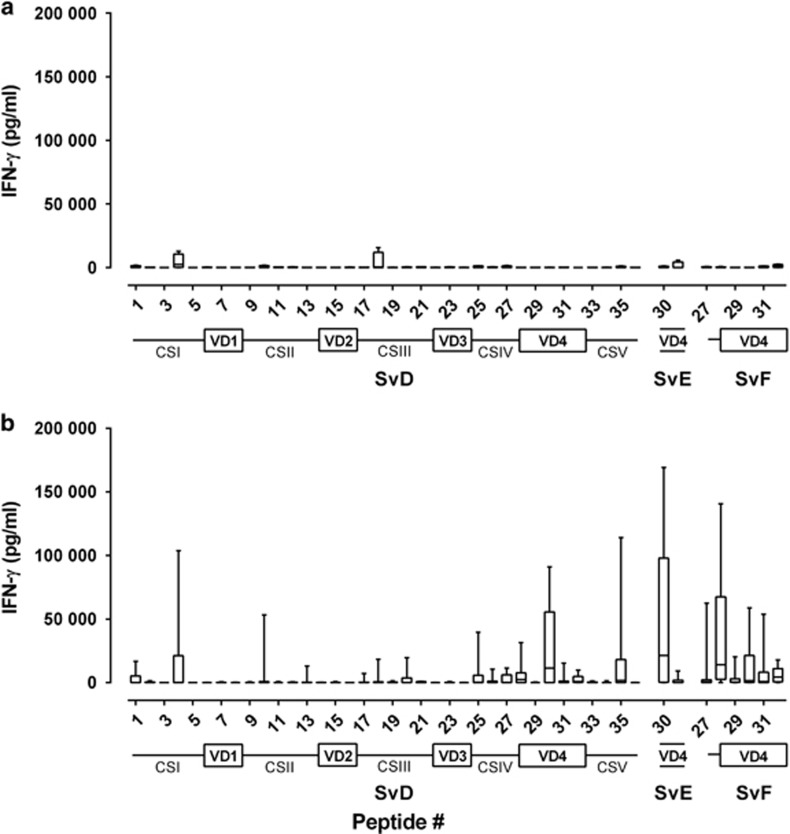
Mapping of T-cell epitopes after two immunizations in peptides (20-mer with 10 aa overlap) covering MOMP. PBMCs from (**a**) UV-SvD/CAF01 (*n*=4)- and (**b**) Hirep1+CTH93/CAF01 (*n*=10)-immunized pigs were restimulated *in vitro* with individual peptides (see Supplementary Table 1 for sequences), spanning the MOMP SvD protein including SvE- and SvF-specific peptides in VD4. The graph is based on the mean IFN-γ value of duplicate culture wells for each pig. The box indicates the 25th and the 75th percentile within the group, the horizontal line within a box represents the median, and whiskers indicate min to max values. The sequence of constant sequences (CS) and VDs of MOMP is depicted below the graphs.

T-cell responses against the individual vaccine components were characterized in more detail by stimulating with peptide pools representing the individual vaccine components (Hirep1, MOMP SvD, CT043 and CT414_aa605-840_) (Supplementary Figure 2). In general, peptide pools representing the individual vaccine components, Hirep1, MOMP SvD and CT043, were frequently and strongly recognized in Hirep1+CTH93/CAF01-vaccinated pigs with levels of IFN-γ significantly different from naïve controls. In contrast, recognition of CT414_aa605-840_ was low. UV-SvD/CAF01-immunized pigs did not respond to peptide pools representing any of the vaccine antigens. To localize T-cell epitopes in the individual components, PBMCs were stimulated with individual 20-mer peptides with 10 aa overlap (Supplementary Tables 1–3) covering the vaccine antigen components (Hirep1 and MOMP SvD (Figure 2), CT043 and CT414_aa605-840_ (Supplementary Figure 3A). For MOMP, peptides covering full length MOMP SvD were used. To cover the Hirep1 fusion construct, peptides covering VD4 of SvE and SvF were included (SvE peptide 30–31, SvF peptide 27–32), when they differed in sequence from VD4 of MOMP SvD. In agreement with the low recognition of the MOMP SvD peptide pools in the UV-SvD/CAF01-vaccinated group, only few T-cell epitopes were detected (Figure 2a). In Hirep1+CTH93/CAF01-immunized pigs, multiple T-cell epitopes were identified throughout MOMP, both in constant and variable domains (Figure 2b). T-cell epitopes were predominantly identified in a region of MOMP (peptide 25–35) comprising conserved sequences and VD4 of SvD, SvE and SvF. Especially, peptide 30 of SvD and SvE, and peptide 28 and 32 of SvF were frequently recognized. The recognition of the Sv-specific peptides, especially SvF peptide 27–32, may indicate a Hirep1-induced T-cell response. In Hirep1+CTH93/CAF01-immunized pigs, the epitopes in CT043 and CT414_aa605-840_ (Supplementary Figure 3A) were recognized by most pigs in peptides 10–13 and 18–23, respectively, whereas the CT043 and CT414_aa605-840_ peptide recognition in UV-SvD/CAF01-immunized pigs was low (data not shown).

### Strong antibody recognition of surface-exposed and Sv-specific regions of MOMP

**Figure 3:**
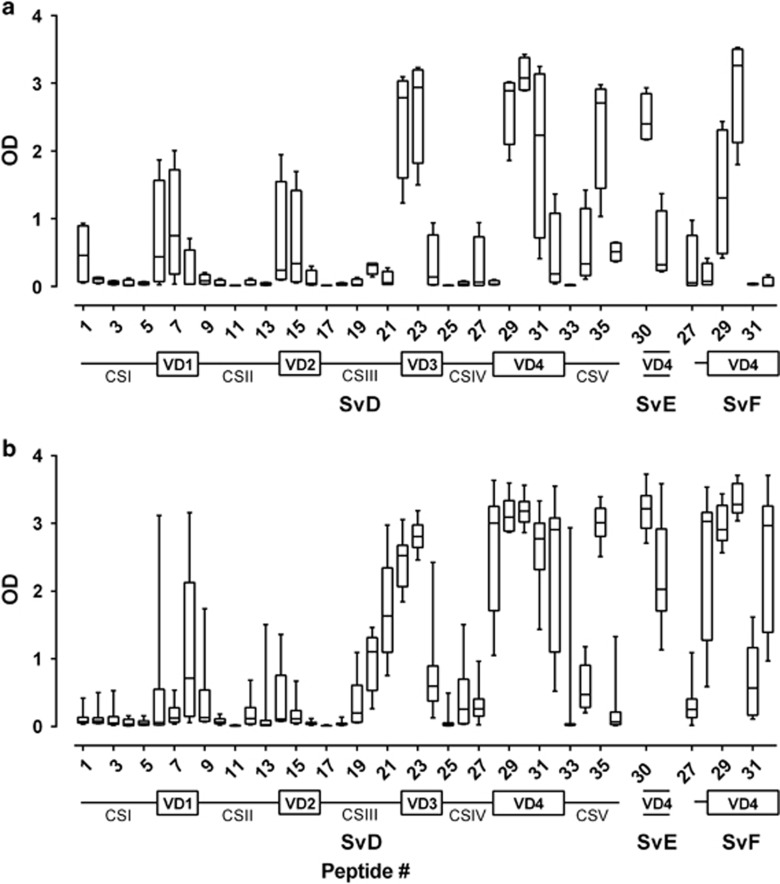
Mapping of B-cell epitopes after two immunizations in peptides (20-mer with 10 aa overlap) covering MOMP. The mapping was performed with sera from (**a**) UV-SvD/CAF01 (*n*=4)- and (**b**) Hirep1+CTH93/CAF01 (*n*=10)-immunized pigs. Serum antibody recognition of overlapping peptides (see Supplementary Table 1 for sequences) spanning MOMP SvD, and SvE- and SvF-specific sequences in VD4. The graph is based on the mean OD value of duplicates for each pig. The box indicates the 25th and the 75th percentile within the group, the horizontal line within a box represents the median, and whiskers indicate min to max values. The sequence of CSs and VDs of MOMP is depicted below the graphs.

To characterize antibody recognition of vaccine components, epitope mapping of linear B-cell epitopes was performed post immunizations (week 7) by evaluating serum antibody recognition of overlapping peptides (20-mer with 10 aa overlap) (Supplementary Tables 1–3) covering the individual vaccine components (MOMP SvD and Hirep1 (Figure 3), CT043 and CT414_aa605-840_ (Supplementary Figure 3B). In UV-SvD/CAF01-immunized pigs, the antibody recognition of MOMP was clustered, largely consistent with the surface-exposed VD sequences (Figure 3a), especially VD3 and VD4. The pattern was similar in Hirep1+CTH93/CAF01-immunized pigs (Figure 3b), but additionally, transmembrane and periplasmic regions adjacent to VD3 and VD4 were recognized. This demonstrates broader antibody recognition owing to presentation of more B-cell epitopes in the hybrid vaccine. Recognition of non-Hirep1 peptides in MOMP SvD (peptide #<25) indicates a CTH93-induced response. In both vaccine groups, peptide 30 of SvD, SvE and SvF was strongly recognized. In addition, peptides of CT043 (peptide 1–2, 6–7 and 14–16) and CT414_aa605-840_ (peptide 1, 4, 14–16 and 22) were strongly recognized in Hirep1+CTH93/CAF01-immunized pigs (Supplementary Figure 3B) compared with low responses in the UV-SvD/CAF01-vaccinated group (data not shown).

### Immune sera neutralizes SvD and SvF infectivity *in vitro*

**Figure 4:**
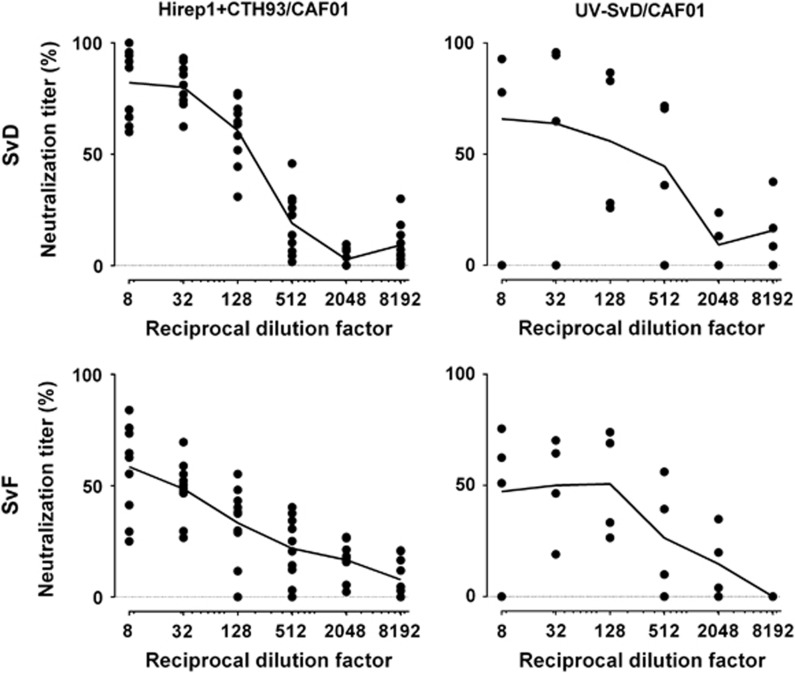
*In vitro* neutralization of *C. trachomatis* SvD and SvF. Serial dilutions of sera from Hirep1+CTH93/CAF01 (*n*=10)- and UV-SvD/CAF01 (*n*=4)-immunized pigs were incubated with SvD or SvF. The sera-bacteria solutions were transferred to HaK cells, and IFUs were fixed, stained and counted. Results are given as the neutralization titer of each immunized pig in relation to pooled sera from naïve controls (*n*=5). Each dot represents the mean of duplicate wells, and a connecting line between the dilutions is shown.

Peptide 30 in MOMP contains the TLNPTIAG sequence (Supplementary Table 1) associated with induction of neutralizing antibodies. As shown in Figure 3, peptide 30 in MOMP SvD, SvE and SvF was strongly recognized by serum antibodies from both Hirep1+CTH93/CAF01 and UV-SvD/CAF01 immunized pigs. Further analysis of sera recognition of peptide 30 in MOMP SvD demonstrated high end point titers in both Hirep+CTH93/CAF01 (median: 125.000, interquartile range: 125.000–125.000)- and UV-SvD/CAF01 (median: 125.000, interquartile range: 35.000–500.000)-immunized pigs. To evaluate the neutralizing abilities of the antibodies, an *in vitro* neutralization assay^32^ was performed using sera collected post immunizations (week 7) and tested against SvD and SvF. We observed strong and broadly neutralizing capacity against SvD and SvF of sera from all individual animals in the Hirep1+CTH93/CAF01 group (Figure 4). Sera from pigs immunized with UV-SvD/CAF01 also had the capability to neutralize SvD and SvF, however, within this group, we observed great variability in responses ranging from no neutralization to as high as observed in the Hirep1+CTH93/CAF01 group.

### Hirep1+CTH93/CAF01 vaccination protects against vaginal infection

**Figure 5:**
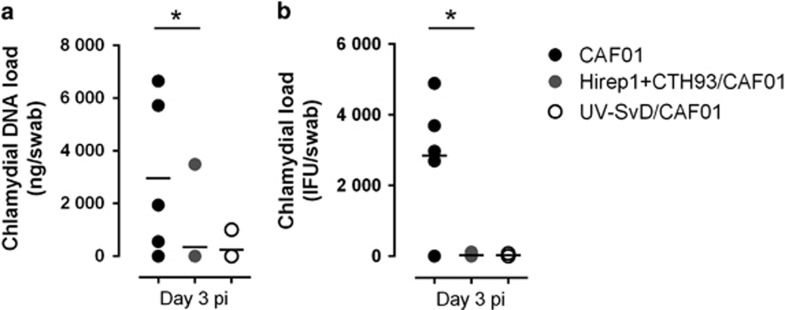
Vaginal loads of *C. trachomatis*. Chlamydial load in CAF01 controls (*n*=5), Hirep1+CTH93/CAF01 (*n*=10)- and UV-SvD/CAF01 (*n*=4)-immunized pigs at day 3 pi was measured in vaginal swabs by (**a**) qPCR (16S rRNA) and (**b**) swab culturing in McCoy cells. In qPCR, each dot represents the mean of triplicates for each pig. In swab culturing, each dot represents one well for each pig. Lines indicate the medians. Statistical difference was calculated using the Kruskal-Wallis test followed by Dunn's multiple comparisons test, *P<0.05.

To evaluate the protective efficacy of the vaccines, the pigs were estrus cycle synchronized and intravaginally inoculated with 3.9 × 10^9^ inclusion forming units (IFUs) during estrus at week 7, and vaginal swabs were taken at days 3, 5, 7 and 12 pi. The levels of chlamydial 16S rRNA were quantified by qPCR, and for comparison at day 3 pi, vaginal swabs were also cultured on McCoy cells to detect the number of IFUs (Figure 5). At day 3 pi, levels of chlamydial DNA and IFUs were significantly decreased in Hirep1+CTH93/CAF01-immunized pigs compared with controls. However, as control pigs rapidly cleared the infection, this protection was only detected at day 3 pi, after which the levels were comparable (data not shown).

### Immune responses post infection demonstrate significant CMI recall in vaccinated pigs

**Figure 6:**
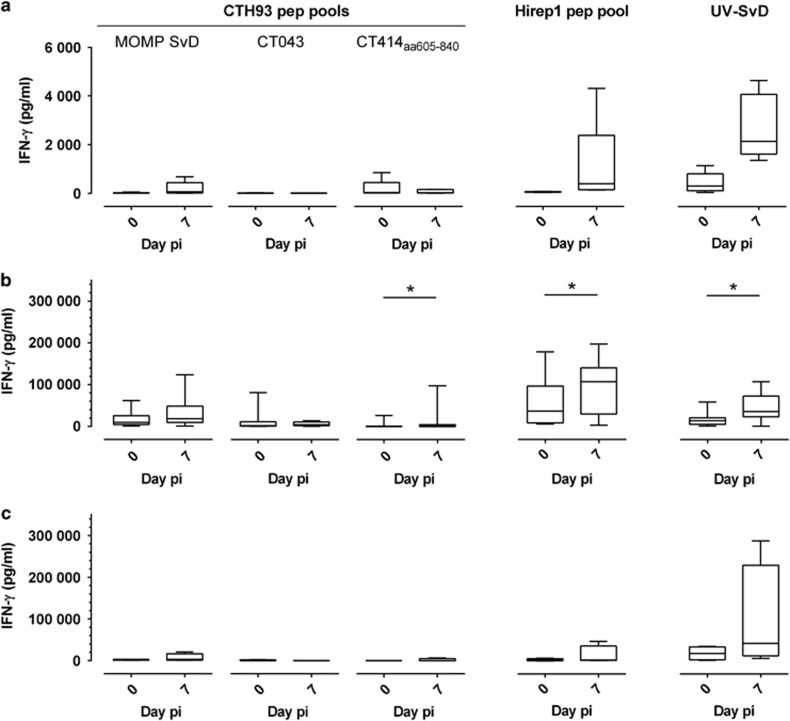
CMI responses post infection. PBMC were stimulated (days 0 and 7 pi) with CTH93 peptide pools (MOMP SvD, CT043 and CT414_aa605-840_), Hirep1 peptide pool and UV-SvD. (**a**) Infected controls (*n*=5), (**b**) Hirep1+CTH93/CAF01-immunized pigs (*n*=10) and (**c**) UV-SvD/CAF01-immunized pigs (*n*=4). For each pig, the mean IFN-γ value of triplicate wells is shown. The box indicates the 25th and the 75th percentile within the group, the horizontal line within a box represents the median, whiskers indicate min to max values. Statistical difference was calculated using the Wilcoxon's matched-pairs rank test, *P<0.05.

Evaluation of antibody responses after the infection was carried out in sera and vaginal swabs against Hirep1, CTH93 and UV-SvD, and results revealed no significant sera and vaginal antibody recall responses in vaccinated pigs (data not shown). Evaluation of CMI responses post challenge was carried out in PBMCs collected at days 0 and 7 pi. CMI responses to the infection alone, represented by pigs immunized with adjuvant alone (CAF01 controls) (Figure 6a), demonstrated UV-SvD-specific IFN-γ responses at day 7 pi (*P*=0.0625), and responses against Hirep1 were also increased (*P*=0.0625). In Hirep1+CTH93/CAF01-vaccinated pigs (Figure 6b), the CMI recall responses against both UV-SvD, Hirep1 and CT414_aa605-840_ was significantly increased, whereas the responses against peptide pools of CT043 and MOMP SvD_aa34-371_ were only modestly elevated. In contrast, the UV-SvD/CAF01 group only responded against UV-SvD (Figure 6c).

## Discussion

Considerable data support the concept that an ideal *Chlamydia* vaccine would need to elicit both humoral and CMI-based immune responses.^33, 34^ Ideally, efficient mucosal neutralizing antibodies reduce initial infectious bacterial load and once intracellular, the bacteria are eliminated by a strong CMI-driven response. Trachoma studies in NHPs and clinical trials in the 1960s using whole-cell vaccines were at least partially successful in reducing conjunctival infection, but protection was short-lived^14^ and Sv-specific;^13^ suggesting that the infection can be controlled by a vaccine if long-term protection and broad Sv coverage are established.

In the present study, we evaluate a multi-subunit vaccine containing Hirep1+CTH93/CAF01. CTH93 is a fusion molecule consisting of three antigens identified in our discovery program: CT414_aa605-840_ (unpublished), CT043 and MOMP SvD_aa34-371._^17, 31, 35^ Hirep1 is a molecularly designed version of MOMP that induces broadly neutralizing antibodies targeting the most prevalent Svs.^29^ To induce a combined humoral and CMI response, we used the CAF01 adjuvant, which has previously been shown to induce both antibody and Th1 cell responses^36^ and has passed clinical safety evaluation.^24^ The vaccines were administered intramuscularly twice, and, regardless of dose (10 or 100 μg), induced highly specific serum and vaginal IgG levels and strong CMI responses (Figure 1). Individual antigens were recognized with a broad spectrum of B- and T-cell epitopes presented.

Epitope mapping of CT043 and CT414_aa605-840_ demonstrated broad B-cell recognition for both, whereas especially CT043 induced a strong and broad T-cell epitope recognition pattern. CT414_aa605-840_ was initially identified in our laboratory in a phage display assay probed with sera from human patients diagnosed with a genital infection (unpublished data) and the full length protein has been shown to react with sera from infected patients,^35^ indicating that the antigen is a B-cell target in infected humans. CT043 was originally identified as a frequent human T-cell antigen^30, 31, 37^ and has later been shown to elicit CD4 T-cell-mediated protection against genital infections in mice, and CT043-specific antibodies recognize the chlamydial surface.^17^

MOMP contains numerous human T-cell epitopes,^38, 39^ consequently aa34–371 of MOMP SvD was included in CTH93 to expand the overall T-cell repertoire. T-cell epitope mapping in MOMP (Figure 2) demonstrated that in pigs, T-cell epitopes are present throughout MOMP with frequently recognized peptides in peptide 25–35 including constant sequence IV, which is known to contain several human T-cell epitopes.^38^ In mice, Hirep1 has been shown to induce both antibody and T-cell responses to Sv-specific and conserved epitopes.^29^ Hirep1-vaccinated mice are protected against genital challenge with *C. trachomatis* SvD and SvF, and this protection is mediated by broadly neutralizing antibodies in concert with a CMI response. Interestingly, we observe multiple vaccine-induced T-cell epitopes recognized in the VD4 region. In particular, peptide 30 in MOMP SvD and SvE containing the conserved neutralizing antibody epitope TLNPTIAG was strongly and frequently recognized. Noticeably, also Sv-specific regions covered in peptides 28, 31 and 32 were recognized. This broad and significant T-cell response covering all Svs represented in the vaccine was unique for the Hirep1+CTH93/CAF01-immunized pigs and not found in the UV-SvD/CAF01 group. Importantly, this Hirep1-specific T-cell response was significantly boosted following the infection (Figure 6), indicating a strong T-cell recall response targeting the VD4 region in MOMP during infection. This broadens the immunological importance of the VD4 region and suggests that beside a clear target for antibodies, this region is also a possible target for T cells during infection. Karunakaran *et al.*^40^ recently suggested that surface proteins, including MOMP, are enriched among the *Chlamydia* T-cell immunoproteome. In that study, mouse MHC class II-bound *C. trachomatis* peptides were identified by proteomics and they identified a peptide, IFDTTTLNPTIAGAGDVK, which is covered in the highly recognized peptide 30 from SvD and SvE used in the present study. Consequently, in line with our observations, this confirms that this region is an important target for vaccine-induced T cells.

Very few MOMP T-cell epitopes were induced in the UV-SvD/CAF01 group, and epitopes recognized were primarily situated within the constant sequences. This is in line with epitope mapping in *C. trachomatis-*infected humans, in which T-cell epitopes of MOMP are mainly located in the CSs,^38, 41^ although also few T-cell epitopes exist in the VDs.^42^

In Hirep1+CTH93/CAF01-immunized pigs, B-cell epitope mapping in MOMP revealed high and frequent antibody recognition of the variable domains and adjacent sequences; in particular, VD3 and VD4 of SvD, and SvE- and SvF-specific sequences of VD4. Sera from Hirep1+CTH93/CAF01-immunized pigs recognized Sv-specific peptides of SvD, SvE and SvF, all surrounding peptide 30 containing the conserved neutralizing epitope TLNPTIAG.^10, 43^ In comparison, the B-cell recognition in UV-SvD/CAF01-immunized pigs showed a narrower epitope pattern, with fewer epitopes recognized, however, still including peptide 30.

The Hirep1+CTH93/CAF01 vaccine induced strong neutralizing antibodies consistently in all animals and sera neutralized both SvD and SvF infection, which confirms our previous findings in mice^29^ and validates the vaccine for future testing in clinical trials. Also, the UV-SvD/CAF01 vaccine induced neutralizing antibodies in line with previously published studies,^19, 20^ but the levels were varying with ranges from not neutralizing to highly neutralizing within the group. Although the UV-SvD/CAF01 group did also recognize peptide 30 encompassing the neutralizing TLNPTIAG epitope, we cannot exclude that other surface proteins may also be involved in induction of neutralizing antibodies, such as PorB^44^ and PmpD^45^ or even conformational epitopes in MOMP. Although with neutralizing potential, antibodies induced by whole-cell vaccines^13^ and derivatives thereof, that is, purified native MOMP, have been shown to promote homotypic immunity in NHPs,^46^ an obstacle that is overcome by the use of recombinant Hirep1 subunit vaccine.

Deep vaginal challenge established an infection that resolved relatively fast and although we observed shedding up to day 12 pi, the levels were very low and no differences between groups were found after day 3 pi (data not shown). Nevertheless, at the very onset on infection (day 3 pi), we did observe protection in both the Hirep1+CTH93 and UV-SvD groups (Figure 5). Given the time point and the neutralizing capacity of sera in both groups, this protection is likely mediated by, at least in part, antibodies. This would also be in line with our previous studies in mice where we observe significant control of bacteria at the early time point (day 3 pi).^29^ Interestingly, we find that Hirep1+CTH93/CAF01-vaccinated pigs mount a significant T-cell recall response directed at peptides representing CT414 and especially Hirep1 indicating that this region is not only a target for antibodies but also for T cells during the infection.

In non-vaccinated controls, the infection induced a T-cell response against UV-SvD. The UV-SvD/CAF01 group had a recall T-cell response against UV-SvD, whereas responses against Hirep1 and CTH93 components were not boosted. In Hirep1+CTH93/CAF01-immunized pigs, CMI responses against Uv-SvD, Hirep1 and CT414_aa605-840_ were significantly increased, demonstrating a recall response against both Hirep1 and CTH93 components (Figure 6). This indicates that although the protective efficacy of the Hirep1+CTH93/CAF01 vaccine and the UV-SvD/CAF01 is equal, the antigen specificity behind this protection might be different.

In conclusion, evaluation of Hirep1+CTH93/CAF01 vaccination in pigs demonstrates a highly immunogenic vaccine with uniform patterns of T- and B-cell epitopes against vaccine components, including high and frequent recognition of SvE- and SvF-specific sequences. Hirep1+CTH93/CAF01 vaccination generates both neutralizing antibodies against SvD and SvF and protective immune responses comparable with the UV-SvD/CAF01 vaccine, but the hybrid vaccine would be a safer alternative, excluding antigens associated with immunopathology.

## Methods

### Ethics statement

The experimental procedures were approved by the Danish Animal Experiments Inspectorate of the Danish Veterinary and Food Administration, license no: 2008/561−1581.

### Chlamydia trachomatis

*C. trachomatis* SvD (Trachoma type D strain UW-3/Cx, ATCC VR-885) was propagated in Hela cells (Human epithelial cell line, Hela 229, ATCC CCL-2.1), and chlamydial elementary bodies (EBs) were harvested and purified as described by Olsen *et al.*^37^ with few modifications: Hela cells were cultured in six-well plates and were infected with 1.5 *C. trachomatis* SvD IFUs per Hela cell. Instead of diethylaminoethyl-dextran treatment, the cells were centrifuged at 750 *g* for 1 h. For use as vaccine antigen, the chlamydial EBs were inactivated by exposure to ultraviolet light emission as described,^47^ and the inactivation step was confirmed by culture in Hela cells. Finally, the content of protein was quantified by the bicinchoninic acid assay, as described in the Micro BCA Protein Assay Kit (cat no 23235, Thermo Scientific, Rockford, IL, USA). For use as the challenge infection, the purified chlamydial EBs were IFU quantified in Hela cells.

### *Chlamydia trachomatis* antigens

Recombinant fusion proteins CTH93 and Hirep1 were produced as follows; CTH93 was designed as a fusion protein of CT043 (full length), CT414_aa605-840_ and MOMP SvD_aa34-371_ (Supplementary Figure 1) with an added N-terminal histidine tag, all sequences obtained from the original annotation published in the study by Stephens *et al.*^48^ The Hirep1 hybrid vaccine was composed of VD4 regions from SvD, SvE and SvF, including residues in the constant regions adjacent to VD4.^29^ Synthetic DNA constructs were codon-optimized for expression in *Escherichia coli* followed by insertion into the pJexpress 411 vector (DNA2.0). To avoid disulfide bridge formation during recombinant production, cysteines in MOMP were exchanged systematically with serines. Purification was carried out essentially as described by Olsen *et al.*^49^

### Vaccines

The hybride antigens were diluted in 1 ml sterile Tris buffer (10 mM, pH 7.4) and were mixed by vortexing with 1 ml cationic liposomic adjuvant CAF01 (500 μg of glycolipid trehalose 6,6′-dibehenate and 2500 μg of dimethyldioctadecylammonium bromide) and sucrose into a total volume of 2 ml isotonic (9% sucrose) vaccine.

### Animals

Nineteen, sexually mature, female Göttingen minipigs were purchased from Ellegaard Göttingen Minipigs A/S, Dalmose, Denmark, which is breeding microbiologically well-defined minipigs in a barrier unit, in which the herds are health-monitored twice a year, following FELASA recommendations. At delivery, the minipigs were 5–6 months old. The pigs were housed in groups of four to six, fed with standard minipig diet twice a day and water *ad libitum*. The study was carried out in a laboratory animal isolation facility at University of Copenhagen.

### Design of study

The 19 female minipigs were randomly divided into four groups immunized with either CAF01, 10 μg Hirep1+CTH93/CAF01, 100 μg Hirep1+CTH93/CAF01 or 125 μg UV-SvD/CAF01; see Table 1. The vaccines were administered twice intramuscularly in the neck muscles, week 0 and week 3. Pigs immunized with CAF01 and UV-SvD/CAF01 are also included in another study focusing on immunohistochemical analysis supported by immunological data and chlamydial shedding. To estrus synchronize the pigs, they were treated orally with 20 mg progestagens (Regumate Equine, MSD Animal Health, Ballerup, Denmark) for 18 days and 6 or 8 days after cessation of treatment, they went into estrus with proximity of a teaser boar. The pigs were sedated with Zoletil mixture (2.5 mg ml^−1^ tiletamine, 2.5 mg ml^−1^ zolazepam, 20 mg ml^−1^ xylazine, 100 mg ml^−1^ ketamin, 10 mg ml^−1^ butorphanol) and inoculated intravaginally at the most cranial part of the vagina with 3.9 × 10^9^ IFUs of *C. trachomatis* SvD diluted in 5 ml SPG (0.2 M sucrose, 20 mM sodium phosphate and 5 mM glutamic acid buffer) with an insemination catheter (Osiris, cat no 902011, E-Vet, Haderslev, Denmark). The pigs were inoculated 30 or 32 days after the second immunization and were killed at day 12 pi by deep anesthesia followed by exsanguination. Rectal temperature and clinical signs were recorded after each vaccination day and on a daily basis after the challenge. Blood samples and vaginal swabs were taken at week 0 and 3 and at days 0, 7 and 12 pi; vaginal swabs were additionally taken at days 3 and 5 pi. For collection of sera, unstabilized blood was centrifuged at 2400 *g* for 15 min, and serum was transferred into new tubes and kept at 20 °C until analysis.

### Vaginal sampling

Before vaginal sampling, the pigs were anesthetized in Zoletil mixture, and the outer vulva was rinsed in water and 70% ethanol. By using a speculum, a swab was introduced into the deeper part of the vagina, close to the cervix, where the swab was rolled clockwise twice along the vaginal wall. The swabs were kept in tubes containing 1 ml SPG. Three autoclaved glass beads were added to each swab sample, followed by whirl mixing for 2 min. The samples were kept at −80 °C until further analysis. On day 3 pi, the swabs were cultured fresh as described below.

### Detection of chlamydial load by culturing

The protocol for detection of chlamydial load in vaginal swabs was modified from the protocol of Hansen *et al.*^50^ Briefly, McCoy cells (murine fibroblast cell line, ATCC CRL-1696) were cultured in infection media (RPMI 1640 supplemented with 5% fetal calf serum, 10 mM HEPES, 1% (vol/vol) non-essential amino acids for MEM Eagle, 2 mM L-glutamine, 1 mM pyrovate, 10 μg ml^−1^ gentamicin, 70 μM mercaptoethanol) in 48-well plates for 24 h. *C. trachomatis* bacteria were detected with sera from MOMP and *C. trachomatis* heat shock protein 60-immunized rabbits followed by staining with 4 μg ml^−1^ Alexa Flour 488-labeled goat-anti rabbit antibody (Cat no: A11008, Life Technologies, Paisley, UK), and McCoy cells were stained with propidium iodide. At least 20 (× 40) fields were counted for IFU in each well using an Olympus IX71 fluorescence microscope (Ballerup, Denmark), and the no of IFUs per swab was calculated.

### Detection of chlamydial load by quantitative PCR

DNA extraction from the vaginal swab samples was performed with Chelex 100 (Bio-Rad, Life Science, Copenhagen, Denmark). One hundred microliters of the swab material were mixed with 300 μl of a 20% Chelex solution in TE buffer (T9285, Sigma Aldrich, Copenhagen, Denmark), vortexed for 60 s and incubated at 96 °C for 10 min. The sample was then centrifuged for 10 min at 17 500 *g* and 4 °C and hereafter triplicates of 5 μl of the supernatant was used for PCR. Real-time qPCR detection of *C. trachomatis* in the vaginal swab samples was performed by detection of the 16S rRNA gene. An internal control (IC) was run in all samples to detect possible inhibition of the PCR. The IC primers were bought from TAG Copenhagen A/S (Copenhagen, Denmark): IC-F 5′ACCGCTCAGGCATTTGCT-3′ and IC-R 5′CCGGGACGTATCATGCT3′. The remaining primers and probes were bought from Applied Biosystems: Ct 16s-F GGATCTTCGGACCTTTCGGT; Ct 16s-R AATCTCTCAATCCGCCTAGACA; Ct 16s-probe FAM-AAGGGAGAGTCTATGTGATAT–MGBNFQ, IC-probe NED-CCTTCGTGATATCGGACGTTGGCTG- MGBNFQ. The assay was performed with: Perfecta qPCR SuperMix (UNG, low ROX, 95066-02K (2000 rx), Quantum Biosciences). The samples were run on a StepOne Real-time PCR instrument (Applied Biosystems) and the instrument was programmed to run 2 min at 95 °C and 40 cycles of denaturation at 95 °C for 15 s and annealing/extension at 60 °C for 1 min. The C_t_ cutoff was determined to be 37, hence C_t_ values greater than 37 were considered as nonsense.

### Measurement of antigen-specific antibodies in serum and in vaginal swabs

Determination of antigen-specific antibodies in serum and vaginal swabs was carried out by indirect ELISA. Ninety-six-well plates were coated overnight with 0.4 μg of UV-SvD (using Polysorp plates, NUNC, Roskilde, Denmark) and 0.1 μg Hirep1, 0.1 μg CTH93 or 0.1 μg MOMP SvD peptide 30 (using Maxisorp plates, NUNC) in bicarbonate/carbonate buffer (pH 9.6) per well. Study samples were added in duplicates in serial dilutions and incubated overnight. An internal standard was used on each plate to correct for plate-to-plate variation. Sample antibodies were detected with horse radish peroxidase-conjugated goat-anti pig IgG secondary antibodies (1:10.000, AAI41P, Serotec, Oxford, UK). The wells were visualized with TMB PLUS substrate (Kem-En-Tec, Taastrup, Denmark) and 0.5 M sulfuric acid. The plates were read at the wavelength of 450 nm with correction at 650 nm. To compensate for plate-to-plate variation, the log-transformed OD mean of the titrated internal standard on each plate was correlated with the log-transformed mean of the internal standard on all plates analyzed simultaneously, resulting in a regression equation for each plate. For each plate, the sample OD values were transformed according to the regression equation. After that, the end point titers were calculated as the highest sample dilution before reaching the cutoff. The cutoff value was calculated for each individual antigen as the mean of the lowest dilution from all week 0 samples added to two times the standard deviation.

B-cell epitope mapping was performed as described for detection of antibodies with few modifications: 96-well Maxisorp plates were coated overnight with duplicates of overlapping peptides (20-mer with 10 aa overlap) (5 μg ml^−1^) covering vaccine antigens (MOMP, Hirep1, CT043 and CT414_aa605-840_). The peptides are listed in Supplementary Tables 1. After the blocking and washing steps, sera drawn after two immunizations (week 7) were diluted (1:200) and added to the wells and serial dilutions of an internal standard were used to minimize plate-to-plate variation, and the plates were incubated overnight and developed as described. The results were given as OD values.

### Measurement of neutralizing antibodies *in vitro*

The neutralizing capacity of the serum antibodies were investigated by an *in vitro* assay, as described.^32^ HaK cells (Syrian Golden hamster epithelial cell line, ATCC CCL-15) were cultured in infection media (as described for culturing swabs in McCoy cells) in Nunclon sterile 96-well plates (Cat no: 167008, Thermo Scientific). *C. trachomatis* SvD or SvF EBs were incubated for 30 min at 37 °C with titrations of heat-inactivated sera from immunized and naïve pigs. EB-sera mixtures were added to the confluent HaK cell layers in duplicates, and SPG and pooled naïve sera from controls were added on each plate as controls. After 2 h of incubation at 35 °C, the EB-sera mix was removed from the wells, and new infection media with 0.5% glucose and 1 μg ml^−1^ cycloheximide were added, followed by incubation at 37 °C for 42 h. *Chlamydia* bacteria were detected with sera from CT043 and cHsp60-immunized rabbits followed by staining with 4 μg ml^−1^ Alexa Flour 488-labeled goat-anti rabbit antibody (Cat no: A11008, Life Technologies), and HaK cells were stained with propidium iodide. At least 20 fields (× 40) were counted for IFUs in each well using an Olympus IX71 fluorescence microscope, and for each dilution, the % inhibition of the sample related to the pooled naïve control sera on the same plate was calculated by the formula: Neutralization %=100—((Mean IFUs of duplicate wells/mean IFUs of pooled sera from naïve controls) × 100).

### *In vitro* stimulation of PBMCs

PBMCs were isolated by density gradient centrifugation on Lympholyte-Mammal medium (1.086 g cm^−3^ at 22 °C, Cedarlane Labs, Skanderborg, Denmark) and cultured in RPMI-1640 with 5 × 10^−5^ M 2-mercaptoethanol, 1 mM glutamine, 1% pyruvate, 1% penicillin-streptomycin, 1% HEPES and 10% fetal calf serum (Invitrogen, Taastrup, Denmark), with 2 × 10^5^ cells/well were added in Nunclon microtiter plates (NUNC). The cells were stimulated in triplicates with UV-SvD, Hirep1, CTH93, CT043, CT414_aa605-840_ and MOMP in 10 μg ml^−1^. Synthetic peptides (20-mer) with 10 aa overlap covering CT043, CT414_aa605-840_, MOMP and Hirep1 were purchased from (Genscript, Piscataway, NJ, USA) and used for stimulation as pools (5 μg ml^−1^ of each peptide in the peptide pools) or as individual peptides (5 μg ml^−1^). The peptides are listed in Supplementary Tables 1. Wells with medium alone and with Staphylococcal enterotoxin B (1 μg ml^−1^) were included as negative and positive control, respectively. After 3 days of incubation at 37 °C with 5% CO_2_, cell culture supernatants were harvested and stored at −20 °C.

### Determination of IFN-γ in cell culture supernatants by ELISA

The levels of IFN-γ in cell culture supernatants were determined by a monoclonal ELISA using anti-porcine IFN-γ monoclonal antibody clone P2F6 (MP700, Thermo Scientific) and biotin-conjugated mouse anti-pig IFN-γ monoclonal antibody clone P2C11 (559958, BD Pharmingen, San Diego, CA, USA), as described by Riber *et al.*^51^ The plates were developed with TMB Plus (Kem-En-Tec) in the dark, and the reaction was stopped by adding 0.5 M sulfuric acid. The OD was read at wavelength 450 nm with correction at 650 nm. From the standards, a double logarithmic curve was constructed to quantify the levels of IFN-γ in the samples.

### Statistics

All statistical analyses were performed using Graph Pad Prism software. To compare time-dependent differences within a group, we used the paired, non-parametric Friedman test followed by Dunn multiple comparisons test. Comparison of difference between selected groups at the same time point was performed by the non-parametric Kruskal-Wallis test followed by Dunn multiple comparisons test. Evaluation of variance within a group at two timepoints was carried out by Wilcoxon's matched-pairs rank test. Significant difference was defined as *P*<0.05 (*P<0.05, **P<0.01, ***P<0.001).

## Figures and Tables

**Table 1:** Design of study

*No of pigs*	*Immunization groups*	*Timepoint*			
		*Week 0*	*Week 3*	*Week 7* *Day 0 post infection*	*Day 12 pi*
5	CAF01	First immunization	Second immunization	Infection	Killing
5	10 μg Hirep1+CTH93/CAF01				
5	100 μg Hirep1 +CTH93/CAF01				
4	125 μg UV-SvD/CAF01				

Abbreviation: pi, post infection.

## References

[bib1] 1World Health OrganizationGlobal incidence and prevalence of selected curable sexually transmitted infections - 2008. World Health Organization. 2012 Report No.: 9789241503839.

[bib2] 2Peipert JF. Clinical practice. Genital chlamydial infections. New Engl J Med 2003; 349: 2424–2430. 14681509 10.1056/NEJMcp030542

[bib3] 3Gottlieb SL, Low N, Newman LM, Bolan G, Kamb M, Broutet N. Toward global prevention of sexually transmitted infections (STIs): the need for STI vaccines. Vaccine 2014; 32: 1527–1535. 24581979 10.1016/j.vaccine.2013.07.087 PMC6794147

[bib4] 4Geisler WM, Lensing SY, Press CG, Hook EW 3rd. Spontaneous resolution of genital *Chlamydia trachomatis* infection in women and protection from reinfection. J Infect Dis 2013; 207: 1850–1856. 23470847 10.1093/infdis/jit094 PMC3654745

[bib5] 5Brunham RC, Kuo CC, Cles L, Holmes KK. Correlation of host immune response with quantitative recovery of *Chlamydia trachomatis* from the human endocervix. Infect Immun 1983; 39: 1491–1494. 6840846 10.1128/iai.39.3.1491-1494.1983 PMC348123

[bib6] 6Cohen CR, Koochesfahani KM, Meier AS, Shen CX, Karunakaran K, Ondondo B et al. Immunoepidemiologic profile of *Chlamydia trachomatis* infection: Importance of heat-shock protein 60 and interferon-gamma. J Infect Dis 2005; 192: 591–599. 16028127 10.1086/432070

[bib7] 7Morrison RP, Caldwell HD. Immunity to murine chlamydial genital infection. Infect Immun 2002; 70: 2741–2751. 12010958 10.1128/IAI.70.6.2741-2751.2002 PMC128027

[bib8] 8Morrison SG, Su H, Caldwell HD, Morrison RP. Immunity to murine *Chlamydia trachomatis* genital tract reinfection involves B cells and CD4(+) T cells but not CD8(+) T cells. Infect Immun 2000; 68: 6979–6987. 11083822 10.1128/iai.68.12.6979-6987.2000 PMC97807

[bib9] 9Su H, Caldwell HD. CD4+ T cells play a significant role in adoptive immunity to *Chlamydia trachomatis* infection of the mouse genital tract. Infect Immun 1995; 63: 3302–3308. 7642259 10.1128/iai.63.9.3302-3308.1995 PMC173455

[bib10] 10Su H, Caldwell HD. Immunogenicity of a synthetic oligopeptide corresponding to antigenically common T-helper and B-cell neutralizing epitopes of the major outer membrane protein of *Chlamydia trachomatis*. Vaccine 1993; 11: 1159–1166. 7504381 10.1016/0264-410x(93)90080-h

[bib11] 11Moore T, Ananaba GA, Bolier J, Bowers S, Belay T, Eko FO et al. Fc receptor regulation of protective immunity against *Chlamydia trachomatis*. Immunology 2002; 105: 213–221. 11872097 10.1046/j.0019-2805.2001.01354.x PMC1782645

[bib12] 12Nichols RL, Bell SD, Murray ES, Haddad NA, Bobb AA. Studies on trachoma: V. clinical observations in a field trial of bivalent trachoma vaccine at three dosage levels in Saudi Arabia. Am J Trop Med Hyg 1966; 15: 639–647. 5941182

[bib13] 13Wang SP, Grayston JT, Alexander ER. Trachoma vaccine studies in monkeys. Am J ophthalmol 1967; 63 (Suppl): 1615–1630. 4960884 10.1016/0002-9394(67)94155-4

[bib14] 14Grayston JT, Wang SP. The potential for vaccine against infection of the genital tract with *Chlamydia trachomatis*. Sex Transm Dis 1978; 5: 73–77. 10328037 10.1097/00007435-197804000-00011

[bib15] 15Coler RN, Bhatia A, Maisonneuve JF, Probst P, Barth B, Ovendale P et al. Identification and characterization of novel recombinant vaccine antigens for immunization against genital *Chlamydia trachomatis*. FEMS Immunol Med Microbiol 2009; 55: 258–270. 19281568 10.1111/j.1574-695X.2008.00527.x PMC2776724

[bib16] 16Finco O, Frigimelica E, Buricchi F, Petracca R, Galli G, Faenzi E et al. Approach to discover T- and B-cell antigens of intracellular pathogens applied to the design of Chlamydia trachomatis vaccines. Proc Natl Acad Sci USA 2011; 108: 9969–9974. 21628568 10.1073/pnas.1101756108 PMC3116399

[bib17] 17Olsen AW, Andersen P, Follmann F. Characterization of protective immune responses promoted by human antigen targets in a urogenital Chlamydia trachomatis mouse model. Vaccine 2014; 32: 685–692. 24365515 10.1016/j.vaccine.2013.11.100

[bib18] 18Rockey DD, Wang J, Lei L, Zhong G. *Chlamydia* vaccine candidates and tools for chlamydial antigen discovery. Expert Rev Vaccines 2009; 8: 1365–1377. 19803759 10.1586/erv.09.98

[bib19] 19Pal S, Theodor I, Peterson EM, de la Maza LM. Immunization with the *Chlamydia trachomatis* mouse pneumonitis major outer membrane protein can elicit a protective immune response against a genital challenge. Infect Immun 2001; 69: 6240–6247. 11553566 10.1128/IAI.69.10.6240-6247.2001 PMC98757

[bib20] 20Sun G, Pal S, Weiland J, Peterson EM, de la Maza LM. Protection against an intranasal challenge by vaccines formulated with native and recombinant preparations of the *Chlamydia trachomatis* major outer membrane protein. Vaccine 2009; 27: 5020–5025. 19446590 10.1016/j.vaccine.2009.05.008 PMC2741729

[bib21] 21Igietseme JU, Eko FO, Black CM. *Chlamydia* vaccines: recent developments and the role of adjuvants in future formulations. Expert Rev Vaccines 2011; 10: 1585–1596. 22043957 10.1586/erv.11.139

[bib22] 22Yu H, Jiang X, Shen C, Karunakaran KP, Jiang J, Rosin NL et al. *Chlamydia muridarum* T-cell antigens formulated with the adjuvant DDA/TDB induce immunity against infection that correlates with a high frequency of gamma interferon (IFN-gamma)/tumor necrosis factor alpha and IFN-gamma/interleukin-17 double-positive CD4+ T cells. Infect Immun 2010; 78: 2272–2282. 20231405 10.1128/IAI.01374-09 PMC2863536

[bib23] 23Karlsson I, Brandt L, Vinner L, Kromann I, Andreasen LV, Andersen P et al. Adjuvanted HLA-supertype restricted subdominant peptides induce new T-cell immunity during untreated HIV-1-infection. Clin Immunol 2013; 146: 120–130. 23314272 10.1016/j.clim.2012.12.005

[bib24] 24van Dissel JT, Joosten SA, Hoff ST, Soonawala D, Prins C, Hokey DA et al. A novel liposomal adjuvant system, CAF01, promotes long-lived Mycobacterium tuberculosis-specific T-cell responses in human. Vaccine 2014; 32: 7098–7107. 25454872 10.1016/j.vaccine.2014.10.036

[bib25] 25Dawson HA comparative assessment of the pig, mouse and human genomes: structural and functional analysis of genes involved in immunity and inflammation. In: McAnulty PAD AD, Ganderup NC, Hastings KL (eds). The Minipig In Biomedical Research. CRC Press: Taylor and Francis Group: Taylor and Francis Group. 2012; pp 323–342.

[bib26] 26Fairbairn L, Kapetanovic R, Sester DP, Hume DA. The mononuclear phagocyte system of the pig as a model for understanding human innate immunity and disease. J Leukoc Biol 2011; 89: 855–871. 21233410 10.1189/jlb.1110607

[bib27] 27Meurens F, Summerfield A, Nauwynck H, Saif L, Gerdts V. The pig: a model for human infectious diseases. Trends Microbiol 2012; 20: 50–57. 22153753 10.1016/j.tim.2011.11.002 PMC7173122

[bib28] 28Vanrompay D, Hoang TQ, De Vos L, Verminnen K, Harkinezhad T, Chiers K et al. Specific-pathogen-free pigs as an animal model for studying *Chlamydia trachomatis* genital infection. Infect Immun 2005; 73: 8317–8321. 16299329 10.1128/IAI.73.12.8317-8321.2005 PMC1307099

[bib29] 29Olsen AW, Follmann F, Erneholm K, Rosenkrands I, Andersen P. Vaccine promoted neutralizing antibodies directed to the VD4 of MOMP protect against *Chlamydia trachomatis* infection and upper genital tract pathology. J Infect Dis e-pub ahead of print 6 March 2015. 10.1093/infdis/jiv137 25748320

[bib30] 30Meoni E, Faenzi E, Frigimelica E, Zedda L, Skibinski D, Giovinazzi S et al. CT043, a protective antigen that induces a CD4+ Th1 response during *Chlamydia trachomatis* infection in mice and humans. Infect Immun 2009; 77: 4168–4176. 19596772 10.1128/IAI.00344-09 PMC2738022

[bib31] 31Follmann F, Olsen AW, Jensen KT, Hansen PR, Andersen P, Theisen M. Antigenic profiling of a *Chlamydia trachomati*s gene-expression library. J Infect Dis 2008; 197: 897–905. 18288899 10.1086/528378

[bib32] 32Byrne GI, Stephens RS, Ada G, Caldwell HD, Su H, Morrison RP et al. Workshop on *in vitro* neutralization of *Chlamydia trachomatis*: summary of proceedings. J Infect Dis 1993; 168: 415–420. 8335979 10.1093/infdis/168.2.415

[bib33] 33Kari L, Bakios LE, Goheen MM, Bess LN, Watkins HS, Southern TR et al. Antibody signature of spontaneous clearance of *Chlamydia trachomatis* ocular infection and partial resistance against re-challenge in a nonhuman primate trachoma model. PLoS Negl Trop Dis 2013; 7: e2248. 23738030 10.1371/journal.pntd.0002248 PMC3667776

[bib34] 34Li W, Murthy AK, Guentzel MN, Chambers JP, Forsthuber TG, Seshu J et al. Immunization with a combination of integral chlamydial antigens and a defined secreted protein induces robust immunity against genital chlamydial challenge. Infect Immun 2010; 78: 3942–3949. 20605976 10.1128/IAI.00346-10 PMC2937460

[bib35] 35Tan C, Hsia RC, Shou H, Haggerty CL, Ness RB, Gaydos CA et al. *Chlamydia trachomatis*-infected patients display variable antibody profiles against the nine-member polymorphic membrane protein family. Infect Immun 2009; 77: 3218–3226. 19487469 10.1128/IAI.01566-08 PMC2715660

[bib36] 36Agger EM, Rosenkrands I, Hansen J, Brahimi K, Vandahl BS, Aagaard C et al. Cationic liposomes formulated with synthetic mycobacterial cordfactor (CAF01): a versatile adjuvant for vaccines with different immunological requirements. PloS One 2008; 3: e3116. 18776936 10.1371/journal.pone.0003116 PMC2525815

[bib37] 37Olsen AW, Follmann F, Jensen K, Hojrup P, Leah R, Sorensen H et al. Identification of CT521 as a frequent target of Th1 cells in patients with urogenital *Chlamydia trachomatis* infection. J Infect Dis 2006; 194: 1258–1266. 17041852 10.1086/508203

[bib38] 38Ortiz L, Demick KP, Petersen JW, Polka M, Rudersdorf RA, Van der Pol B et al. *Chlamydia trachomatis* major outer membrane protein (MOMP) epitopes that activate HLA class II-restricted T cells from infected humans. J Immunol 1996; 157: 4554–4567. 8906834

[bib39] 39Arno JN, Xie C, Jones RB, Van Der Pol B. Identification of T cells that respond to serovar-specific regions of the *Chlamydia trachomatis* major outer membrane protein in persons with serovar E infection. J Infect Dis 1998; 178: 1713–1718. 9815224 10.1086/314478

[bib40] 40Karunakaran KP, Yu H, Jiang X, Chan Q, Moon KM, Foster LJ et al. Outer membrane proteins preferentially load MHC class II peptides: implications for a *Chlamydia trachomatis* T cell vaccine. Vaccine 2015; 33: 2159–2166. 25738816 10.1016/j.vaccine.2015.02.055 PMC4390527

[bib41] 41Kim SK, DeMars R. Epitope clusters in the major outer membrane protein of *Chlamydia trachomatis*. Curr Opin Immunol 2001; 13: 429–436. 11498298 10.1016/s0952-7915(00)00237-5

[bib42] 42Ortiz L, Angevine M, Kim SK, Watkins D, DeMars R. T-cell epitopes in variable segments of *Chlamydia trachomatis* major outer membrane protein elicit serovar-specific immune responses in infected humans. Infect Immun 2000; 68: 1719–1723. 10678996 10.1128/iai.68.3.1719-1723.2000 PMC97337

[bib43] 43Peterson EM, Cheng X, Markoff BA, Fielder TJ, de la Maza LM. Functional and structural mapping of *Chlamydia trachomatis* species-specific major outer membrane protein epitopes by use of neutralizing monoclonal antibodies. Infect Immun 1991; 59: 4147–4153. 1718870 10.1128/iai.59.11.4147-4153.1991 PMC259009

[bib44] 44Kubo A, Stephens RS. Characterization and functional analysis of PorB, a *Chlamydia* porin and neutralizing target. Mol Microbiol 2000; 38: 772–780. 11115112 10.1046/j.1365-2958.2000.02167.x

[bib45] 45Kari L, Southern TR, Downey CJ, Watkins HS, Randall LB, Taylor LD et al. *Chlamydia trachomatis* polymorphic membrane protein D is a virulence factor involved in early host-cell interactions. Infect Immun 2014; 82: 2756–2762. 24733093 10.1128/IAI.01686-14 PMC4097629

[bib46] 46Kari L, Whitmire WM, Crane DD, Reveneau N, Carlson JH, Goheen MM et al. *Chlamydia trachomatis* native major outer membrane protein induces partial protection in nonhuman primates: implication for a trachoma transmission-blocking vaccine. J Immunol 2009; 182: 8063–8070. 19494332 10.4049/jimmunol.0804375 PMC2692073

[bib47] 47Rey-Ladino J, Koochesfahani KM, Zaharik ML, Shen CX, Brunham RC. A live and inactivated *Chlamydia trachomatis* mouse pneumonitis strain induces the maturation of dendritic cells that are phenotypically and immunologically distinct. Infect Immun 2005; 73: 1568–1577. 15731055 10.1128/IAI.73.3.1568-1577.2005 PMC1064943

[bib48] 48Stephens RS, Kalman S, Lammel C, Fan J, Marathe R, Aravind L et al. Genome sequence of an obligate intracellular pathogen of humans: *Chlamydia trachomatis*. Science 1998; 282: 754–759. 9784136 10.1126/science.282.5389.754

[bib49] 49Olsen AW, Follmann F, Hojrup P, Leah R, Sand C, Andersen P et al. Identification of human T cell targets recognized during *Chlamydia trachomatis* genital infection. J Infect Dis 2007; 196: 1546–1552. 18008235 10.1086/522524

[bib50] 50Hansen J, Jensen KT, Follmann F, Agger EM, Theisen M, Andersen P. Liposome delivery of *Chlamydia muridarum* major outer membrane protein primes a Th1 response that protects against genital chlamydial infection in a mouse model. J Infect Dis 2008; 198: 758–767. 18652549 10.1086/590670

[bib51] 51Riber U, Boesen HT, Jakobsen JT, Nguyen LT, Jungersen G. Co-incubation with IL-18 potentiates antigen-specific IFN-gamma response in a whole-blood stimulation assay for measurement of cell-mediated immune responses in pigs experimentally infected with *Lawsonia intracellularis*. Vet Immunol Immunopathol 2011; 139: 257–263. 20889217 10.1016/j.vetimm.2010.09.001

