# Supplementary material for: A multi-subunit Chlamydia vaccine inducing neutralizing antibodies and strong IFN-γ+ CMI responses protects against a genital infection in minipigs
Source: Immunol Cell Biol. 2015 Sep 22;94(2):185–95. doi: 10.1038/icb.2015.79 (PMC4748142; doi:10.1038/icb.2015.79)
Supplement: Supplementary Information [file icb201579x1.pdf]

Supplementary information

Supp. Table 1 MOMP peptides

Peptide no	AA no	AA residues[1]	Peptides covering VD regions[2]
**SvD**			
1	1-20	LPVGNPAEPSLMIDGILWEG	
2	11-30	LMIDGILWEGFGGDPCDPCA	
3	21-40	FGGDPCDPCATWCDAISMRV	
4	31-50	TWCDAISMRVGYYGDFVFDR	
5	41-60	GYYGDFVFDRVLKTDVNKEF	
6	51-70	VLKTDVNKEFQMGAKPTTDT	VD1
7	61-80	QMGAKPTTDTGNSAAPSTLT	VD1
8	71-90	GNSAAPSTLTARENPAYGRH	VD1
9	81-100	ARENPAYGRHMQDAEMFTNA	
10	91-110	MQDAEMFTNAACMALNIWDR	
11	101-120	ACMALNIWDRFDVFCTLGAT	
12	111-130	FDVFCTLGATSGYLKGNSAS	
13	121-140	SGYLKGNSASFNLVGLFGDN	
14	131-150	FNLVGLFGDNENQKTVKAES	VD2
15	141-160	ENQKTVKAESVPNMSFDQSV	VD2
16	151-170	VPNMSFDQSVVELYTDTTFA	VD2
17	161-180	VELYTDTTFAWSVGARAALW	
18	171-190	WSVGARAALWECGCATLGAS	
19	181-200	ECGCATLGASFQYAQSKPKV	
20	191-210	FQYAQSKPKVEELNVLCNAA	
21	201-220	EELNVLCNAAEFTINKPKGY	
22	211-230	EFTINKPKGYVGKEFPLDLT	VD3
23	221-240	VGKEFPLDLTAGTDAATGTK	VD3
24	231-250	AGTDAATGTKDASIDYHEWQ	VD3
25	241-260	DASIDYHEWQASLALSYRLN	
26	251-270	ASLALSYRLNMFTPYIGVKW	
27	261-280	MFTPYIGVKWSRASFDADTI	
28	271-290	SRASFDADTIRIAQPKSATA	VD4
29	281-300	RIAQPKSATAIFDTTTLNPT	VD4
30	291-310	IFDTTTLNPTIAGAGDVKTG	VD4
31	301-320	IAGAGDVKTGAEGQLGDTMQ	VD4

32	311-330	AEGQLGDTMQIVSLQLNKMK	VD4
33	321-340	IVSLQLNKMKSRKSCGIAVG	
34	331-350	SRKSCGIAVGTTIVDADKYA	
35	341-360	TTIVDADKYAVTVETRLIDE	
36	351-371	VTVETRLIDERAAHVNAQFRF	
**SvE**			
30	291-310	IFDTTTLNPTIAGAGDVK**AS**	VD4
31	301-320	IAGAGDVK**AS**AEGQLGDTMQ	VD4
**SvF**			
27	262-281	MFTPYIGVKWSRASFD**S**DTI	
28	272-291	SRASFD**S**DTIRIAQP**RLVTP**	VD4
29	282-301	RIAQP**RLVTPVV**D**I**TTLNPT	VD4
30	292-311	**VV**D**I**TTLNPTIAG**CGS**V**AGA**	VD4
31	302-322	IAG**CGS**V**AGANT**EGQ**IS**DTMQ	VD4
32	313-332	**T**EGQ**IS**DTMQIVSLQLNKMK	VD4

[1] The amino acid residues of the VD regions in MOMP SvD, E and F are given as in[53]. The VD regions are highlighted in grey, and the TLNPTIAG sequence is underlined. Amino acid residues in the extended VD4 region specific for MOMP SvE and SvF are marked in bold.

[2] Peptides covering four or more aa in VD regions are defined as VD peptides.

Supp. Table 2 CT043 peptides

Peptide no	AA no	AA residues
1	1-20	MSRQNAEENLKNFAKELKLP
2	11-30	KNFAKELKLPDVAFDQNNTC
3	21-40	DVAFDQNNTCILFVDGEFSL
4	31-50	ILFVDGEFSLHLTYEEHSDR
5	41-60	HLTYEEHSDRLYVYAPLLDG
6	51-70	LYVYAPLLDGLPDNPQRRLA
7	61-80	LPDNPQRRLALYEKLLEGSM
8	71-90	LYEKLLEGSMLGGQMAGGGV
9	81-100	LGGQMAGGGVGVATKEQLIL
10	91-110	GVATKEQLILMHCVLDMKYA
11	101-120	MHCVLDMKYAETNLLKAFAQ
12	111-130	ETNLLKAFAQLFIETVVKWR
13	121-140	LFIETVVKWRTVCSDISAGR
14	131-150	TVCSDISAGREPTVDTMPQM
15	141-160	EPTVDTMPQMPQGGGGGIQP
16	151-167	PQGGGGGIQPPPAGIRA

Supp. Table 3 Peptides of CT414 $_{aa605-840}$

Peptide no	AA no in full length protein	AA residues
1	605-624	TVKAIVESTPEAPEEIPPVE
2	615-634	EAPEEIPPVEGEESTATEDP
3	625-644	GEESTATEDPNSNTEGSSAN
4	635-654	NSNTEGSSANTNLEGSQGDT
5	645-664	TNLEGSQGDTADTGTGDVNN
6	655-674	ADTGTGDVNNESQDTSDTGN
7	665-684	ESQDTSDTGNAESEEQLQDS
8	675-694	AESEEQLQDSTQSNEENTLP
9	685-704	TQSNEENTLPNSNIDQSNEN
10	695-714	NSNIDQSNENTDESSDSHTE
11	705-724	TDESSDSHTEEITDESVSSS
12	715-734	EITDESVSSSSESGSSTPQD
13	725-744	SESGSSTPQDGGAASSGAPS
14	735-754	GGAASSGAPSGDQSISANAC
15	745-764	GDQSISANACLAKSYAASTD
16	755-774	LAKSYAASTDSSPVSNSSGS
17	765-784	SSPVSNSSGSEEPVTSSSDS
18	775-794	EEPVTSSSDSDVTASSDNPD
19	785-804	DVTASSDNPDSSSSGDSAGD
20	795-814	SSSSGDSAGDSEEPTEPEAG
21	805-824	SEEPTEPEAGSTTETLTLIG
22	815-834	STTETLTLIGGGAIYGETVK
23	825-840	GGAIYGETVKIENFSG

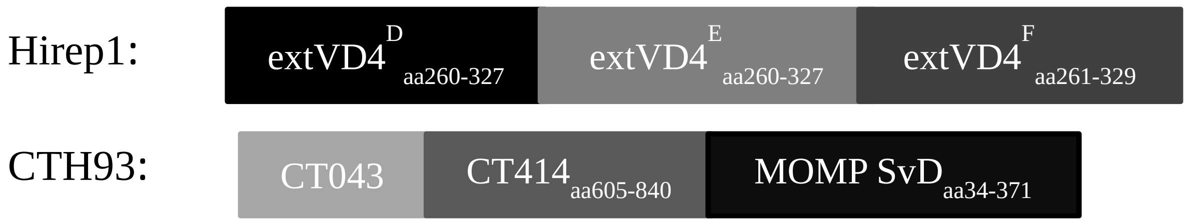

Supp. Figure 1 **Illustration of the antigen components in Hirep1 and CTH93**. Hirep1 consists of extended (ext) VD4 regions from MOMP SvD, SvE and SvF. CTH93 consists of CT043, CT414 $_{aa605\text{-}840}$ and MOMP SvD$_{aa34\text{-}371}$.

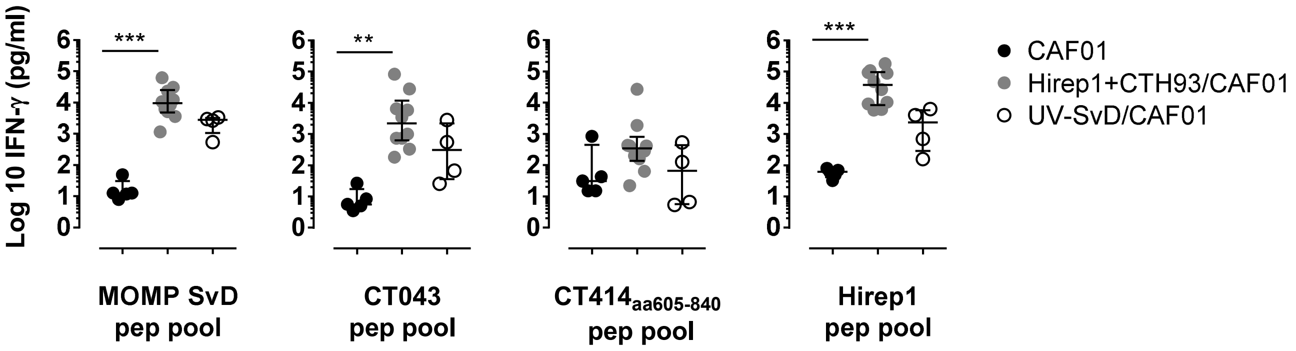

Supp. Figure 2 **Characterization of CMI responses against peptide pools four weeks after the second immunization (week 7).** PBMCs from CAF01 controls (n= 5), Hirep1+CTH93/CAF01 (n=10) and UV-SvD/CAF01 (n=4) immunized pigs were stimulated in triplicates with pools of peptides covering (**a**) MOMP SvD, (**b**) CT043, (**c**) CT414 $_{aa605\text{-}840}$ and (**d**) Hirep1. Each dot represents the mean of triplicate culture wells for each pig, and lines indicate medians ± interquartile range within a group. Kruskal-Wallis test followed by Dunn’s multiple comparisons test, ** $p<0.01$, *** $p<0.001$.

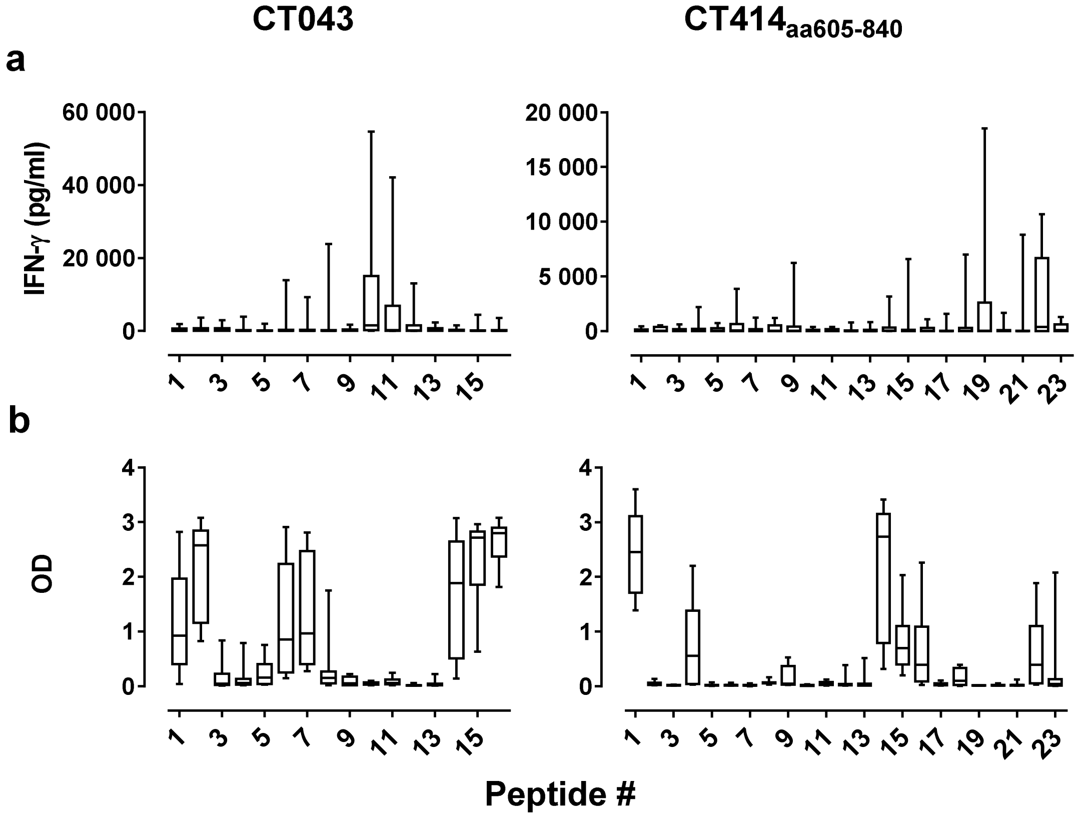

Supp. Figure 3 Epitope mapping in peptides spanning CT043 and CT414$_{aa605-840}$ in Hirep1+CTH93/CAF01 immunized pigs (n=10), four weeks post the second immunization (Week 7). The peptides (20-mers with 10aa overlap) are listed in Supp. Tables 2-3. (**a**) T cell epitope mapping was performed by restimulating PBMCs *in vitro* with overlapping peptides. The graph is based on the mean value of duplicate culture wells for each pig. The box indicates the 25th and the 75th percentile within the group, the horizontal line within a box represents the median, and whiskers indicate min to max values. (**b**) B cell epitope mapping was performed by evaluating sera antibody recognition of overlapping peptides. The graph is based on the mean value of duplicate wells for each pig. The box indicates the 25th and the 75th percentile within the group, the horizontal line within a box represents the median, and whiskers indicate min to max values.
